# The Composition and Differentiation of the Seed-Associated Microbiome in Rapeseed Seeds as Studied Through 218 Rapeseed Transcriptomes

**DOI:** 10.3390/ijms27135801

**Published:** 2026-06-26

**Authors:** Lei Sheng, Yu Wang, Peicheng Lu, Guomin Han, Zhongping Hao, Shumin Hou

**Affiliations:** 1 Bio-breeding Laboratory of Anhui Province, Crop Research Institute, Anhui Academy of Agricultural Sciences, Hefei 230036, China; 13685514362@163.com (L.S.);; 2 The National Key Engineering Lab of Crop Stress Resistance Breeding, The School of Life Sciences, Anhui Agricultural University, Hefei 230036, China

**Keywords:** rapeseed, transcriptome data, seed-associated microbiome, composition, differentiation

## Abstract

Rapeseed is one of the most important oil crops in the world. Its yield and quality are severely restricted by biotic stress and abiotic stress. Rapeseed seeds play a crucial role in the propagation process, and the microorganisms in the seeds can be vertically passed on to the next generation, which greatly affects the quality, yield and growth of rapeseed. However, from a group perspective, there is currently a lack of systematic research on the composition of seed-associated microbiome within rapeseed seeds. This study utilized the transcriptome data of 218 rapeseed seeds that have been published, focusing on analyzing and comparing the dynamic changes and functional differences in the composition of seed-associated microbiome in rapeseed seeds under normal growth and development, biologic stress and abiotic stress conditions. Since we used public transcriptome data without surface sterilisation control, we refered to the detected microorganisms as seed-associated microbiome. The advantage of this study lies in its application of this method to a large-scale sample of rapeseed populations, which systematically revealed the response characteristics of seed-associated microbiome under different stress conditions. Interestingly, some widely distributed genera were not detected, while rare taxa were found under specific conditions, warranting further verification. Since these microorganisms originated from the seeds, their compatibility with plants and colonization ability may far exceed those of soil-derived agents. In the future, high-throughput screening of strains with excellent antagonistic or repellent effects against major diseases and pests of rapeseed can be conducted from these unique seed-associated microbiome. These strains that were confirmed by culture-based, amplicon or metagenomic approaches can then be used to develop seed coating agents or soil inoculants.

## 1. Introduction

Microorganisms are the most abundant and diverse biological resources on Earth [1]. Among them, endophytes are microorganisms that live asymptomatically inside plant tissues. Plant endophytes are mainly represented by bacteria and fungi, while archaebacteria, algae, protozoa, viruses and nematodes are rarely found to be living as endophytes [2]. Endophytic microorganisms are commonly found in the roots [3], stems [4], leaves [5], flowers [6], fruits [7], seeds [8], and other tissues of plants. In recent years, a large number of endophytic microorganisms have been isolated from the storage organs of *Cucumis sativus*, beetroot, *Brassica oleracea*, peanut kernels, the roots and leaves of corn, the stems of *Bermuda* grass, tubers, the seeds and ovules of potato, the radicles of cotton, cotton bolls, the leaves of rice and other plants. By comparing different organs and tissues of plants, it can be found that different endophytic microorganisms occupy different ecological niches [9]. For a particular plant, the endophytic microorganisms that can be isolated from it range from several to dozens, and some even reach hundreds [10]. Currently, it has been reported that more than 129 types of plant endophytic bacteria have been discovered in various crops and economic plants, belonging to 54 genera [11].

Endophytic microorganisms can establish a relatively stable symbiotic and synergistic relationship with plants and play a variety of roles in plants, such as nitrogen fixation, siderophore, stress resistance, and the promotion of phosphorus and potassium absorption [12,13,14,15]. For example, the endophytic nitrogen-fixing bacteria in sugarcane and rice provide them with sufficient nitrogen [16]. Wang et al. isolated an acid-producing *Klebsiella oxytoca* strain from rice, which can secrete auxin and promote the growth of rice. This strain can significantly enhance the growth and development of plants [17]. Furthermore, plants possess multiple mechanisms to resist external biological or non-biological stressors. Among these, the research on how plants’ endophytic microorganisms produce secondary metabolites to withstand adverse conditions has gradually become a hot topic in current endophytic microbe studies [18]. For example, Tian et al. studied the significant role of the antifungal proteins secreted by the endophyte *Epichloe festucae* in controlling leaf blight [19]. In 2014, Lahlali et al. detected through qPCR that the colonization of the dark-spored endophyte *Heteroconium chaetospira* in the roots of rapeseed was negatively correlated with the symptoms of root rot disease [20]. The endophytic *Sporobolomyces ruberrimus* isolated from a serpentine population of Arabidopsis arenosa protected plants against excess metals [21]. Aleynova et al. described the influence of the grapevine bacterial and fungal endophytes on stilbene production in grape cells [22]. Pitakbut et al. discovered the synthesis of cytotoxic macrolide maytansine by endophytes inside their plant host and also reported the impact of maytansine production on plant secondary metabolites [23].

In recent years, with the development of microbiome research, the potential role of endophytic microorganisms in seeds in crop growth, stress resistance and quality formation has received increasing attention. Studies have reported the composition and functions of seed endophytic bacteria using culture methods or amplicon sequencing techniques in crops such as corn, rice and wheat [24,25]. However, the traditional culture methods or amplicon sequencing techniques that are commonly used in studies can reveal the species composition of microorganisms, but they are unable to reflect the transcriptional activity of endophytic bacteria in seeds and their real-time responses to the host’s physiological state and environmental stress [26]. Rapeseed is one of the most important oil crops in the world. Its yield and quality are severely restricted by biological stress and abiotic stress. At present, there is a lack of large-scale and population-level comparative studies on the endophytic bacterial communities in rapeseed under various stress conditions. In particular, there is a need to analyze the dynamic changes of the seed-associated microbiome at the natural population level and their relationships with the growth, quality and stress resistance of rapeseed. Chen et al. developed a new analysis strategy to obtain endophytic microbiome information from plant transcriptome data. Based on the principle that plant transcriptome data also contain endophytic microbiome data, ribosome-coding sequences are distinguished from protein-coding sequences in transcriptome data, and the composition information of archaea, bacteria, fungi, viruses, protozoa, and other species with ribosomal coding sequences can be obtained [27,28]. Therefore, this study utilized the published transcriptome data of 218 rapeseed seeds, focusing on analyzing and comparing the dynamic changes and functional differences in the composition of the seed-associated microbiome in rapeseed seeds under normal growth and development as well as biological stress and abiotic stress conditions, looking forward to their future application potential in green agriculture. Considering that the data used in this study are public transcriptome data, it cannot be confirmed whether the original samples have undergone surface sterilization treatment, we refer to the microorganisms detected in this study collectively as the seed-associated microbiome rather than specifically referring to the endophytic microbiome.

## 2. Results

### 2.1. Seed-Associated Microbiome Composition in the 218 Rapeseed Samples

**Figure 1:**
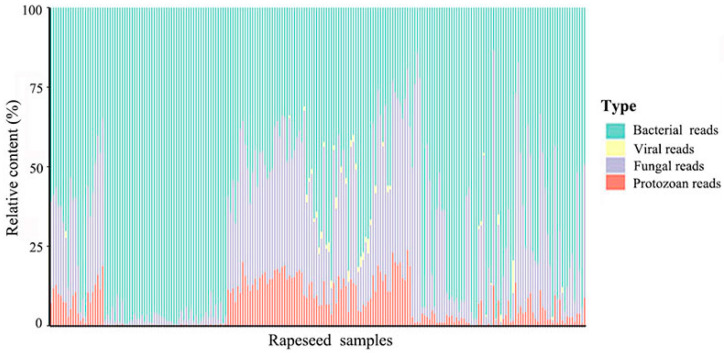
Relative content of bacteria, fungi, protozoa, and viruses in 218 samples.

The cleaned reads were separated into files containing rRNA and those without rRNA using SortMeRNA software. The proportion of rRNA in each sample ranged from 0.13% to 75.74% (Table S2). The wide range of rRNA proportions in various samples might be related to several factors. Firstly, the developmental stage of seeds has a significant impact on endogenous rRNA abundance. Mature seeds in an active metabolic state have a higher rRNA transcription level, while seeds entering dormancy or senescence stages have relatively lower rRNA content. Secondly, there were differences in RNA integrity among different seed samples. Some samples might show a decrease in rRNA proportion due to RNA degradation during sampling, preservation, or extraction processes. Thirdly, the sequencing library construction strategy and sequencing depth also affect the detection proportion of rRNA read sequences. Species annotation and classification showed that the proportion of *Brassica napus transcript* abundance ranged from 1.70% to 82.81%, while the proportion of microbial genome content ranged from 17.19% to 98.30% (Table S2). Among the microorganisms present in each sample, fungi and bacteria were usually the most abundant, followed by protozoa, with viruses being the least abundant (Figure 1, Table S3).

**Figure 2:**
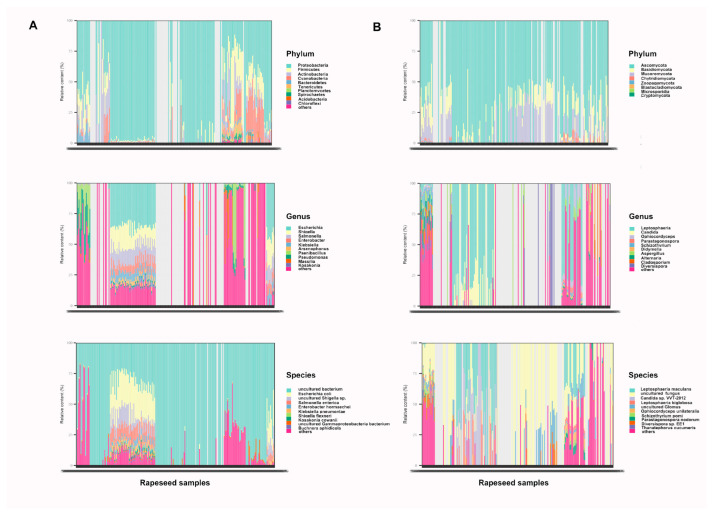
Community composition of bacteria and fungi in 218 samples. (**A**) Community composition of bacteria of top 10 abundance at the phylum, genus and species level. (**B**) Community composition of fungi of top 10 abundance at the phylum, genus and species level.

Among the 218 rapeseed samples, only 12 samples were found to contain archaea, and the abundance of archaea in each sample was relatively low. Bacteria were detected in 180 samples, with a total of 25 phyla, 312 genera, and 532 species identified. Fungi were detected in 165 samples, distributed across 8 phyla, 209 genera, and 273 species. There were significant differences in the relative abundance of genera and species among different rapeseed samples (Figure 2).

### 2.2. Seed-Associated Microbiome Composition in Rapeseed Samples for Different Periods

**Figure 3:**
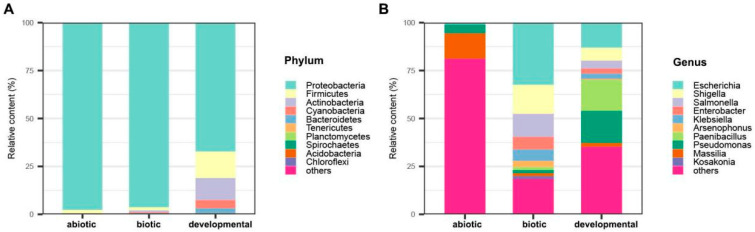
Analysis of composition in different groups of bacteria communities at the phylum level (**A**) and the genus level (**B**).

Based on the original SRA metadata and sample annotation information, this study divided 218 rapeseed transcriptome samples into a developmental group, biotic group and abiotic group. The samples of developmental group were classified according to seed development stage-related experiments, including samples from different developmental periods such as flowering, 7 days after flowering, 10 days after flowering, 14 days after flowering, 45 days after flowering, and the germination period. In addition, this group also included experimental treatments related to seed traits, such as brown seeds, high harvest index, low harvest index, seed coat color, polyploidization and transgene overexpression. The samples of the biotic group mainly originated from pathogen inoculation experiments of the *Leptosphaeria* genus, including samples of *Leptosphaeria* maculans and *Leptosphaeria biglobosa* treatments, with treatment times including 72 h, 96 h, 168 h, 264 h, 336 h, etc. Corresponding un-inoculated control samples were also included. The samples of the abiotic group mainly originated from low-temperature-treatment experiments and their corresponding control samples. Their growth period was the germination period. At the phylum level, the dominant bacteria in the bacterial communities of the abiotic group and biotic groups belonged to *Proteobacteria* and *Firmicutes*, while the dominant bacteria in the bacterial communities of the developmental group belonged to *Proteobacteria*, *Firmicutes*, *Actinobacteria*, *Cyanobacteria*, and *Bacteroidetes* (Figure 3A). At the genus level, the overall bacterial community structure among the three groups varied significantly. In the abiotic group, the dominant genera were *Massilia* and *Pseudomonas*, with *Massilia* accounting for more than 13% of the overall microbial abundance. In the biotic group, the dominant genera were *Escherichia*, *Shigella*, and *Salmonella*, with *Escherichia* accounting for more than 38% of the overall microbial abundance. In the developmental group, the dominant genera were *Pseudomonas*, *Paenibacillus* and *Escherichia*, with *Pseudomonas* accounting for more than 15% of the overall microbial abundance (Figure 3B).

**Figure 4:**
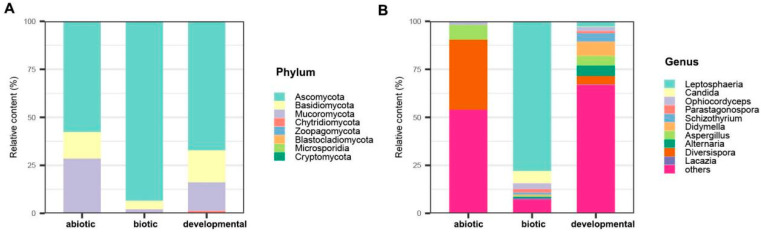
Analysis of composition in different groups of fungi communities at the phylum level (**A**) and the genus level (**B**).

The fungal community composition at the phylum level exhibited similarities among the three groups, with differences in relative abundance. The dominant species in the biotic group belonged to *Ascomycota*, while the dominant species in the abiotic group and developmental groups belonged to *Ascomycota*, *Mucoromycota*, and *Basidiomycota*. In all the groups, *Ascomycota* had the highest relative abundance, accounting for more than 63%, 93%, and 67% (Figure 4A). At the genus level, the dominant species in the abiotic group belonged to *Diversispora*. The dominant species in the biotic group belonged to *Leptosphaeria*, and the dominant species in the developmental group belonged to *Didymella*. In the biotic group, *Leptosphaeria* had the highest relative abundance, accounting for more than 78%, while the abiotic and developmental groups had very low abundances. The abiotic group had a significantly higher relative abundance of the genus *Diversispora* compared to the other groups (Figure 4B).

**Figure 5:**
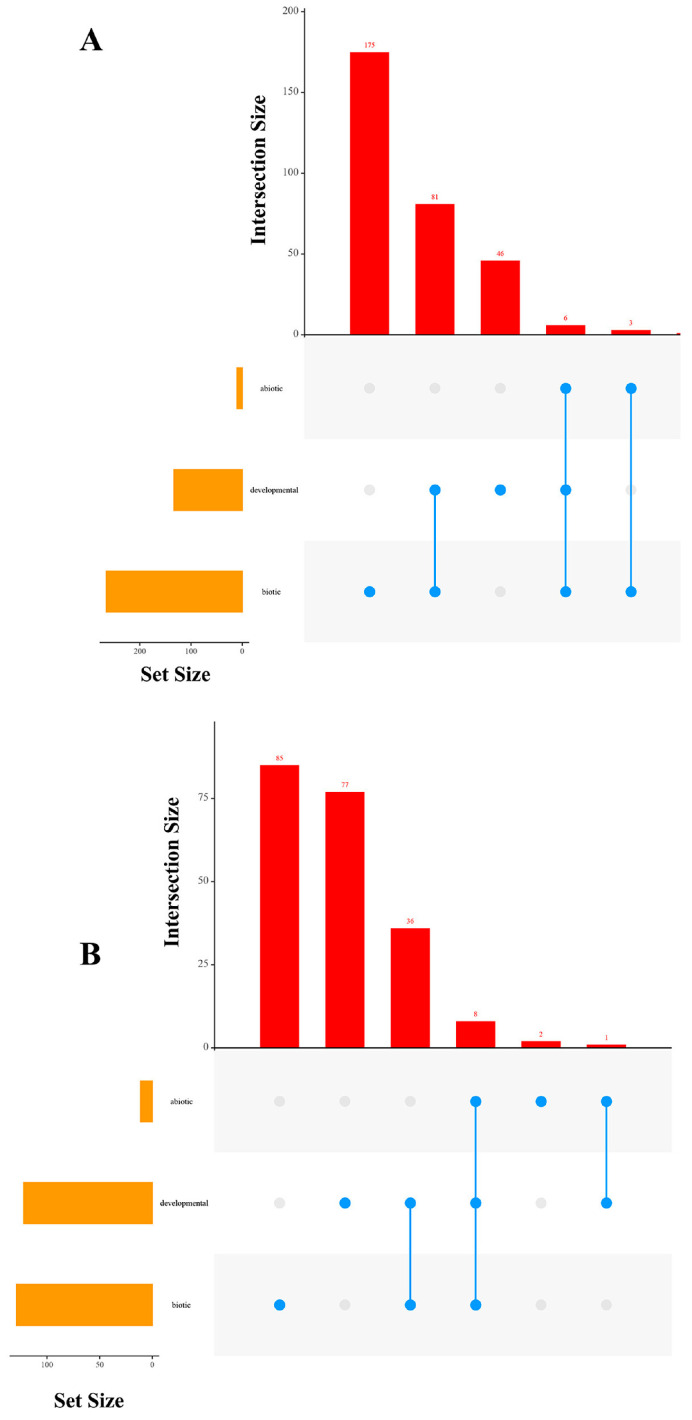
Common and unique genera in different groups of bacteria (**A**) and fungi (**B**).

To further explore the commonalities among the groups, an abundance upset plot analysis was conducted at the genus level. The upset analysis at the genus level showed that there were six bacterial genera shared among the three groups, accounting for 30%, 2.26%, and 4.51% of the total bacterial genera in the abiotic, biotic, and developmental groups, respectively. Additionally, each group had its own unique bacterial genera, with 1, 175, and 46 genera in the abiotic, biotic, and developmental groups, respectively, representing 5%, 66.04%, and 34.59% of the total bacterial genera in each group (Figure 5A). The upset analysis also showed that there were eight fungal genera shared among the three groups, accounting for 72.73%, 6.20%, and 6.56% of the total fungal genera in the abiotic, biotic, and developmental groups, respectively. Additionally, each group had its own unique fungal genera, with 2, 85, and 77 genera in the abiotic, biotic, and developmental groups, respectively, representing 18.18%, 65.89%, and 63.11% of the total fungal genera in each group (Figure 5B).

### 2.3. Seed-Associated Microbiome Diversity in Rapeseed Samples for Different Periods

**Figure 6:**
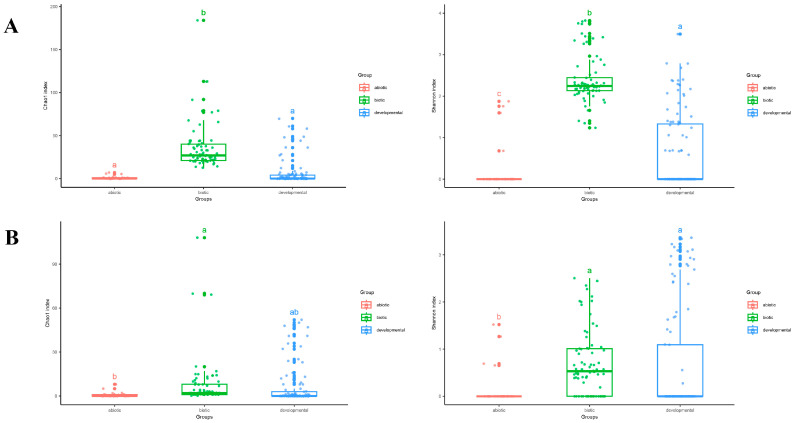
Alpha diversity analysis of microorganisms in different groups. (**A**) Bacteria alpha diversity analysis in different groups, including violin boxplot of the *Chao1* index and *Shannon* index. (**B**) Fungi alpha diversity analysis in different groups, including violin boxplot of the *Chao1* index and *Shannon* index.

The *Chao1* index and the *Shannon* index are two of the most commonly used α-diversity indices in ecology and microbiomics. They describe the species diversity within a community (sample) from different perspectives. The *Chao1* index is used to estimate the total number of microbial species existing in a community, including those rare species that have not been detected at the current sequencing depth. The *Shannon* index is used to comprehensively evaluate the species richness and evenness within a community. When analyzing microbial communities, a high *Chao1* index indicates a rich variety of species types, while a high *Shannon* index suggests a complex, stable community structure and potentially greater functional diversity. *Alpha* diversity analysis showed that there were no significant differences in the *Shannon* index among the different groups for bacteria, and the difference in the *Chao1* index among the different groups was also not significant (Figure 6A). As for fungi, there were no significant differences in the *Chao1* index among the different groups. However, there were significant differences in the *Shannon* index among the different groups (*p* < 0.01), indicating significant differences in fungal diversity at the genus level among the different groups (Figure 6B).

**Figure 7:**
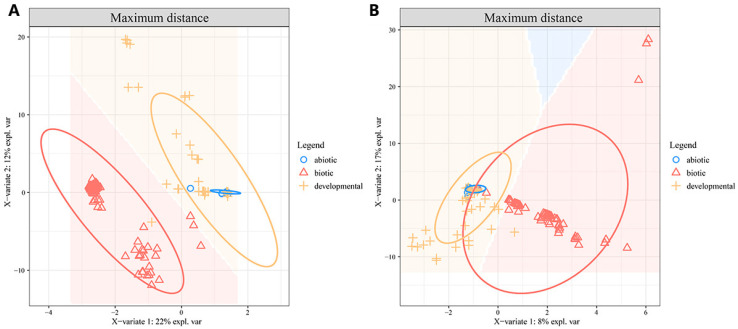
PLS-DA analysis of microorganisms at the genus level in different groups. (**A**) PLS-DA analysis of bacteria at the genus level in different groups. (**B**) PLS-DA analysis of fungi at the genus level in different groups.

PLS-DA was used to perform β-diversity analysis at the genus level for bacteria and fungi. The results showed that in the bacterial community, X-variate1 and X-variate2 accounted for 1.22% and 2.12% of the variation, respectively (Figure 7A). In fungal communities, X-variate1 accounted for 1.8% of the variation, while X-variate2 accounted for 2.17% of the variation. The PLS-DA analysis showed that the first two components explained less than 4% of the total variation in the bacterial community, but in this low-dimensional space, there was a partial separation trend between the biotic group, the development group, and the abiotic group. The biotic group showed significant differences when compared with the abiotic group and the developmental group (Figure 7B).

### 2.4. Species Difference Analysis for Different Periods

**Figure 8:**
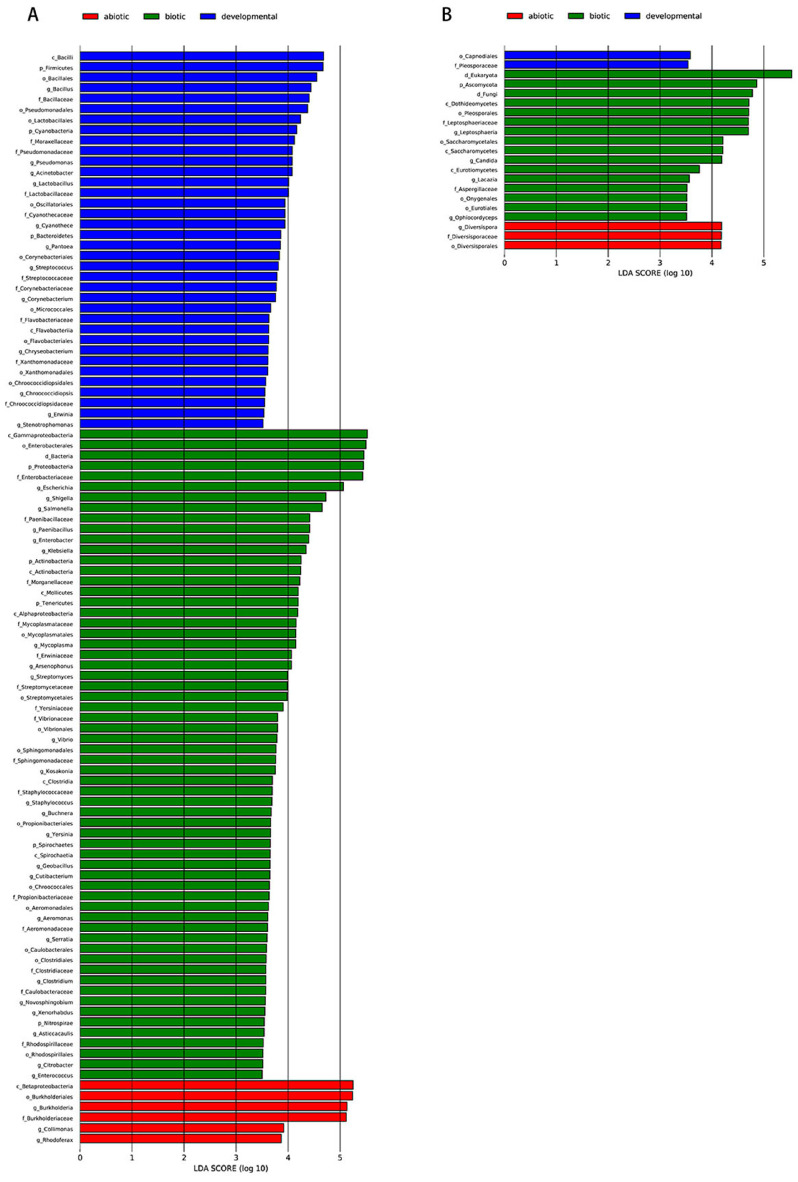
Distribution of LDA value of bacterial species (**A**) and fungal species (**B**) in different groups.

To further explore the species with significant differences in bacteria and fungi among the three groups, LEFSe analysis was conducted. In bacteria, at the phylum level, Firmicutes, Cyanobacteria, and Bacteroidetes were identified as characteristic microbial taxa for the developmental group, while *Proteobacteria*, *Actinobacteria*, *Tenericutes*, *Spirochaetes* and *Nitrospirae* were identified as characteristic microbial taxa for the biotic group when the LDA score was greater than 3.5. At the genus level, *Burkholderia*, *Collimonas*, and *Rhodoferax* were significantly enriched in the abiotic group. *Bacillus*, *Pseudomonas*, *Acinetobacter*, *Lactobacillus*, *Cyanothece*, and *Pantoea* were significantly enriched in the developmental group. The biotic group had the most significantly enriched species, such as *Escherichia*, *Shigella*, *Salmonella*, *Paenibacillus*, *Enterobacter*, *Klebsiella*, *Mycoplasma* and *Arsenophonus*. In fungi, at the phylum level, *Ascomycota* was significantly enriched in the biotic group. At the genus level, *Leptosphaeria*, *Candida*, *Lacazia*, and *Ophiocordyceps* were significantly enriched in the biotic group, while *Diversispora* was significantly enriched in the abiotic group (Figure 8).

### 2.5. Research on the Symbiotic Network of Microorganisms in Rapeseed Samples for Different Periods

**Figure 9:**
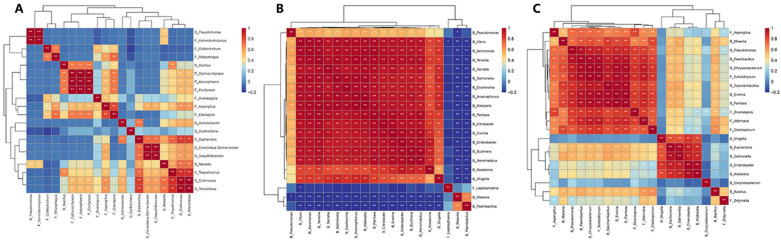
Heatmap of correlations of relative abundance of top 20 species in different groups. (**A**) Heatmap of correlations of relative abundance of top 20 species in abiotic group. (**B**) Heatmap of correlations of relative abundance of top 20 species in biotic group. (**C**) Heatmap of correlations of relative abundance of top 20 species in developmental group. Note: B_means bacterial genus, F_means fungal genus, V_means viral genus, * *p* < 0.05, ** *p* < 0.01.

After calculating the *Spearman* correlation coefficients between each pair of species in the abiotic group, the biotic group, and the developmental group, we selected the 20 species with the highest relative abundance in each of the three groups to construct a heatmap (Figure 9). This provided an initial understanding of the interactions among species. Under abiotic stress, the dominant microbial species mainly adapt to the physical and chemical environment rather than each other. They form a loose, fragile, and environment-tolerance-oriented “survival-type” community with weak interspecies interactions. Under biotic stress, the dominant species form a “combat-type” community with the core function of cooperative defense, jointly resisting external enemies. The plant development stage shapes a dynamic, stable, and mild synergy and is closely coupled with the plant physiological state “symbiosis-type” community. The correlation is stronger overall than that of the abiotic group but weaker than that of the biotic group, and it is dominated by a weak positive correlation.

**Figure 10:**
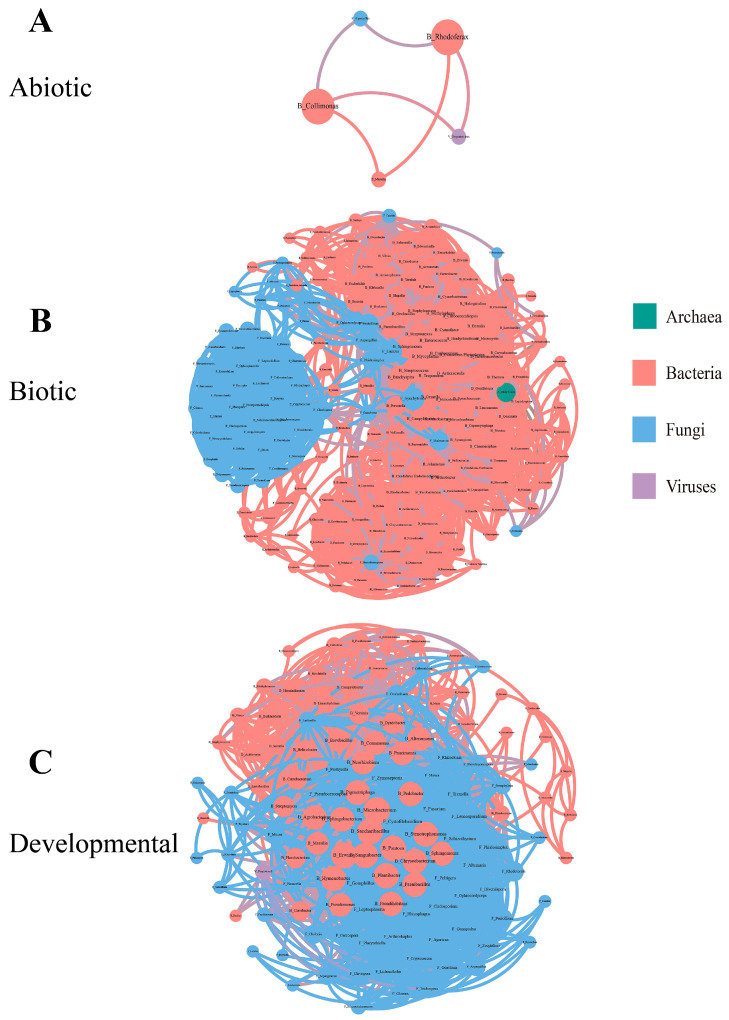
Co-occurrence network of bacteria–fungi at the genus level in the seed of different rapeseed samples. (**A**) Co-occurrence network in the abiotic group. (**B**) Co-occurrence network in the biotic group. (**C**) Co-occurrence network in the developmental group. Note: red indicates bacteria, and blue indicates fungi.

Cytoscape 3.9.1 software was used to construct the co-occurrence network of bacteria and fungi at the genus level for rapeseed samples in different periods (Figure 10). The results showed that the networks for different periods exhibited distinct morphological differences. The network of the abiotic group consisted of only five species, namely *F_Aspergillus*, *B_Massilia*, *B_Collimonas*, *B_Rhodoferax*, and *V_Tequatrovirus*. The microbial communities in the biotic group and the developmental group were very complex and more interconnected, mainly consisting of bacterial–fungal interactions. Under abiotic stress conditions, such as high temperature, drought, and salt damage, most of the microorganisms were likely filtered out, leaving only a few groups having strong adaptive physiological mechanisms or capable of surviving in extreme microhabitats, such as *F_Aspergillus* (fungus of the *Aspergillus* genus). Many *Aspergillus* species are renowned for their ability to produce antioxidant molecules and osmotic regulatory substances and to stabilize cell structures. They are known as pioneer microorganisms for resisting adverse conditions such as drought and salinity. The common feature of the biotic group and the developmental group is their high complexity and close connection, which reflects that microorganisms have formed complex nutritional exchanges, signal communications, and competitive cooperative relationships, enabling the community as a whole to possess resilience against pathogen invasion and environmental fluctuations. Bacteria–fungi interactions are a typical feature of soil and plant microbial communities. Bacteria and fungi form the “framework” of the ecological network and participate in core ecosystem functions such as carbon and nitrogen cycling, nutrient mobilization, and hormone regulation [29]. Under biotic stress, the interactions tend to focus on constructing defense alliances. During the growth and development stages, the interaction network is organized around nutrient supply and growth regulation, thus meeting the needs of specific developmental stages such as seed development and grain filling.

The interactions between microbial species can be either positive, promoting symbiosis and symbiotic relationships, or negative, involving inhibition and competition. The prevalence of positive interactions in the three groups was higher than that of negative interactions, indicating that the degree of cooperation among microbial genera was greater than that of confrontation. In the abiotic group and the developmental group, 100% of the interactions were positive. In the biotic group, 86.63% of the interactions were positive, while 13.37% were negative (Table S4). Comparing the basic network information and topological parameters, it can be clearly seen that the overall positive interaction ratios of the biotic group and the developmental group networks played a dominant role, but their interaction structures had qualitative differences. One contained antagonistic relationships, while the other had none. The abiotic group showed a significantly different result compared to the other two groups (Table S5). The degree distributions of the biotic group and the developmental group followed a power-law distribution, indicating that they possessed the characteristic of scale-free networks. The average path length in the networks ranged from 1 to 3. The biotic group and the developmental group networks exhibited higher values than the abiotic group network in terms of average degree and average weighted degree. Similarly, in terms of network diameter, modularity, and average path length, the values of the biotic group and the developmental group networks were also higher than those of the abiotic group network. Furthermore, the average clustering coefficients of the biotic group and the developmental group networks were similar, while the average clustering coefficient of the abiotic group was 0. However, the abiotic group network exhibited higher values than the biotic group and the developmental group networks in terms of graph density. These results indicated that the microbial aggregation and proximity in the networks of the biotic group and the developmental group were higher than those in the abiotic group, and the biotic group and the developmental group had more complex and tighter connections.

### 2.6. Modular Analysis of Microorganisms in Rapeseed Samples for Different Periods

**Figure 11:**
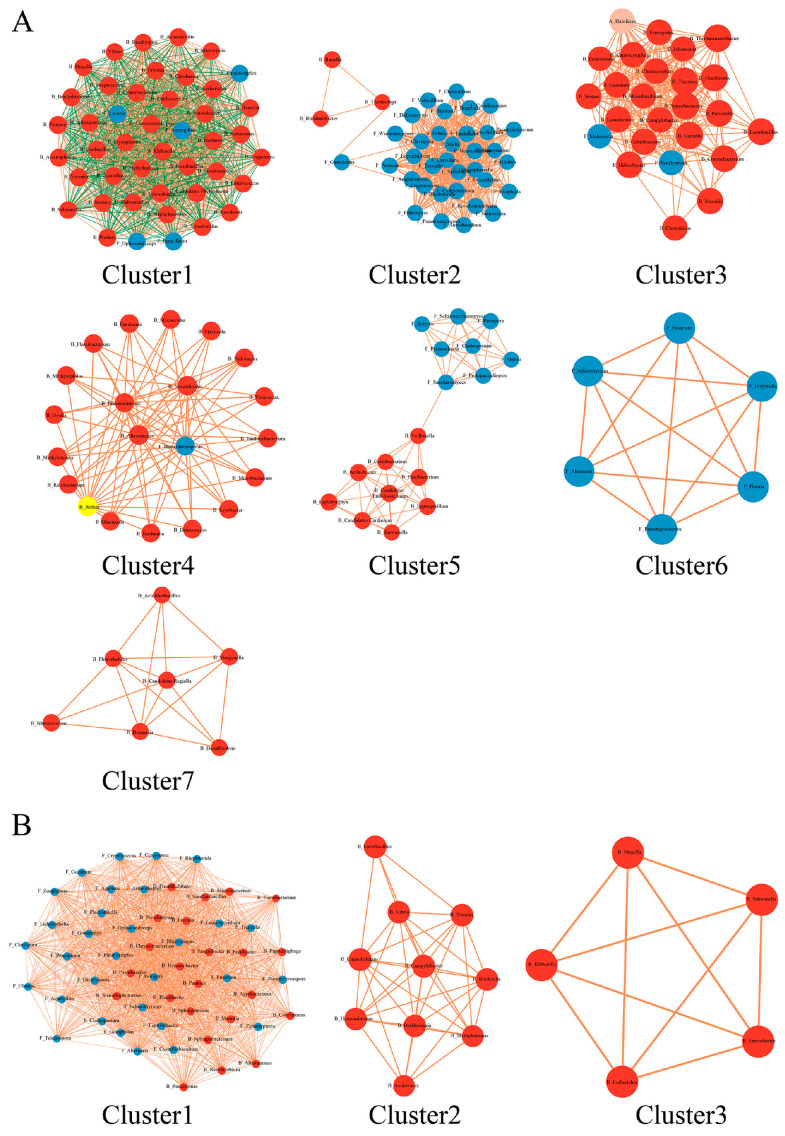
Strong relationship module of the biotic group (**A**) and the developmental group (**B**). Note: Red is bacteria, blue is fungi, red edge is positive correlation, and green edge is negative correlation.

The network module was detected using the MCODE algorithm in Cytoscape 3.9.1. In the biotic group’s network, thirteen sub-network modules were identified by consensus, among which seven were recognized as closely related modules (score > 5.0). Similarly, in the developmental group’s network, seven subnetwork modules were detected, among which three were classified as closely related modules (score > 5.0) (Table S6, Figure 11). There was only one module for the abiotic group, and the sub-network module was not identified. Modules are highly interconnected regions in the network, typically composed of microbial species with similar functions that promote each other; they also reflect the aggregation of related species, niche overlap, and coevolution among species [30]. We analyzed the species information of the top three scoring modules in the biotic group and the developmental group networks (Table S7). In cluster 1 of the biotic group network, 23 belonged to bacterial genera and 31 belonged to fungal genera among all 54 nodes. In cluster 2, three belonged to bacterial genera and 34 belonged to fungal genera among all 37 nodes. In cluster 3, 22 belonged to bacterial genera, two belonged to fungal genera, and one belonged to archaeal genera among all 25 nodes. Cluster 1 of the developmental group network was exactly the same as cluster 1 of the biotic group network. In cluster 2 of the developmental group network, all 10 nodes were bacterial genera. In cluster 3, all five nodes were also bacterial genera. From the above analysis, it can be seen that the species composition of each module was highly specific.

## 3. Discussion

This study, based on 218 transcriptome data of rapeseed, initially revealed the basic composition and differentiation patterns of the seed-associated microbiome community in rapeseed seeds. The results showed that fungi and bacteria were the core components of the microbial community in seeds, and their relative abundances were significantly higher than those of protozoa and viruses. This finding is consistent with the general rules of plant microbiomes: bacteria and fungi often occupy a dominant position in the plant microecosystem due to their metabolic diversity, strong environmental adaptability, and long-term co-evolution history with the host. Fungi, as important decomposers and symbiotic partners, may provide key nutrients during the early stage of seed germination, while bacteria play a significant role in biological nitrogen fixation, hormone regulation, and disease resistance. Viruses are relatively rare, but they may still be important factors regulating the dynamics of the microbial community, especially in mediating horizontal gene transfer or regulating host–microbe interactions.

We investigated the differences in the composition of seed-associated microbiome communities in rapeseed during its development process, as well as under biotic and abiotic stress treatments. At the genus level, the overall bacterial community structure among the three groups varies significantly. The dominant genera of the abiotic group was *Massilia*, the dominant genera of the biotic group was *Escherichia*, and the dominant genera of the developmental group was *Pseudomonas*. *Pseudomonas* played a dominant role during the developmental stage, which was consistent with the existing related reports that the endophytic bacteria of the *Pseudomonas* genus isolated from reeds were found to be able to promote the growth of seedlings and the development of roots and stems and to stimulate the formation of root hairs when inoculated onto rice, *Bermuda* grass, and annual bluegrass [31,32]. These differences may reflect the active filtering and targeted selection mechanisms of endophytic microbe communities within plant seeds under different ecological selection pressures.

Plants can vertically transfer specific advantageous microbial groups to their offspring through seeds. This transfer is not a simple passive carrying process but rather a “selective bottleneck” process regulated by the host’s genetics. The fungal community composition at the phylum level exhibited similarities among the three groups, with differences in relative abundance. At the genus level, the dominant species in the abiotic group belonged to *Diversispora*, the dominant species in the biotic group belonged to *Leptosphaeria*, and the dominant species in the developmental group belonged to *Didymella*. In the biotic group, *Leptosphaeria* had the highest relative abundance, accounting for more than 78%, while the abiotic and developmental groups had very low abundances. The significant enrichment of *Leptosphaeria* at the genus level in the biotic stress group, as well as the dominance of *Diversispora* in the abiotic stress group, indicated that rapeseed selectively accumulated microorganisms with specific protective functions when facing different types of stress. Previously, *Padmathilake* and *Fernando* had already reported that less virulent *Leptosphaeria* biglobosa immunizes the canola plant to resist highly virulent *L. maculans* [33]. The abiotic group had a significantly higher relative abundance of the genus *Diversispora* compared to the other groups, corresponding to the previous relevant literature reports that the inoculation of the arbuscular mycorrhizal fungus *Diversispora* eburnea had an impact on the growth of *Lolium* perenne and *Amorpha* fruticosa, as well as on cadmium absorption and the distribution of cadmium in cadmium-contaminated soil [34].

The upset analysis revealed that there were common and unique microbial species among the abiotic, biotic, and developmental groups, with the biotic group containing the largest number of unique species. When rapeseed plants are subjected to biological stressors such as pathogens and pests, their seeds “enrich” or “select” a more diverse and unique microbial species. These microorganisms are not randomly present but are actively recruited by the plants under stress or passed on to the next generation as a “symbiotic defense alliance”. The endophytic bacteria in the seeds have the characteristic of vertical transmission, allowing beneficial traits to be directly passed on to the next generation. The microbial diversity of seeds is highest under stress, indicating that plants may store the “best stress-resistant microbial team” accumulated throughout their lives in the seeds, providing the first line of defense for their offspring. The alpha diversity shows significant differences among the groups, with the biotic group having the highest diversity. Higher alpha diversity usually means a richer functional gene pool and stronger community stability. In the face of complex stressors, diverse microbial communities can protect the host plants through more diverse mechanisms.

Analyzing the symbiotic network of microorganisms showed that the positive interactions among microorganisms in the three groups were more prevalent than negative interactions, indicating that the degree of cooperation among microbial genera was higher than the degree of competition. Under abiotic stress, the symbiotic network of microorganisms became extremely simplified (consisting of only five nodes), and the community structure was fragile. The retained groups, such as the *Aspergillus* fungi (*F_Aspergillus*) and bacterial genera like *Massilia*, *Collimonas*, and *Rhodoferax*, have all been reported to possess stress-resistant mechanisms such as drought tolerance, salt tolerance, or antioxidant properties [35,36,37]. This simplified network structure does not represent random collapse. Rather it is a strategic reduction in the community, where plants preferentially retain microorganisms that directly resist abiotic stress by producing osmotic regulatory substances, antioxidant molecules, or stress-resistant structures, thereby maintaining the minimum level of symbiotic functions in extreme environments. The seed-associated microbiome in rapeseed exhibited a highly complex and closely connected interaction network during the growth and development stages and under biotic stress, with bacterial–fungal interactions forming the network framework. This structural feature reflected the functional integration and ecological resilience of the microbial community, as well as the core position of bacterial–fungal interactions in the plant microbial ecosystem. The two interact through cross-border signal communication, metabolic complementarity, or physical association to form functional modules, jointly maintaining the stability and functional output of the community.

The modular analysis results showed significant differences in the species composition of different modules, reflecting functional division refinement. For instance, cluster 3 of the biotic group network consisted mainly of bacteria and was likely to be responsible for the core metabolic cycle, while cluster 2 was dominated by fungi and may be responsible for organic matter degradation or symbiotic structure formation. Another interesting phenomenon was that cluster 1 of the developmental group network was exactly the same as cluster 1 of the biotic group network. This phenomenon indicated the existence of a core, stable microbial cooperative alliance whose composition and interaction patterns were not influenced by the classification methods of the biotic group or developmental group. This core module may perform fundamental and essential ecological functions such as carbon and nitrogen cycles and the synthesis of primary metabolites, and it is the basis for maintaining the system’s functions.

This study, from an ecological perspective, revealed the assembly patterns and functional differentiation of the seed-associated microbiome in rapeseed under different environmental conditions, providing a new perspective for understanding the interaction between plants and microorganisms. The candidate core microorganisms selected under abiotic stress can potentially serve as microbial inoculants in the future and be used to enhance the survival ability of rapeseed under adverse conditions. The existence of complex networks under biotic stress suggests that is possible to enhance the systemic resistance of plants to diseases and reduce the reliance on chemical pesticides by regulating the structure of the microbial community. The functional specificity of the microbial network at the growth and development stages of seed indicates that applying growth-promoting microbial preparations during critical growth stages may be more effective in enhancing yield and quality. In terms of application prospects, we could identify the microbial nodes that play a key role in the resistance, tolerance or growth promotion of rapeseed and carry out a series of studies based on the network analysis results of this study. For example, LEfSe analysis can be used to screen out candidate core microbial markers with significant differential abundance from the biotic stress group and the abiotic stress group, especially those endophytic fungal or bacterial groups that are highly enriched under stress conditions and positively correlated with host health. Then, these candidate core strains can be isolated and purified from the rapeseed or tissue of the corresponding treatment groups, and a cultivable strain resource library can be established. Through in vitro antagonistic tests and sterile seed inoculation systems, the antibacterial activity of candidate strains against major rapeseed diseases and their effects on the growth of rapeseed seedlings can be verified one by one. Further, excellent single strains or synthetic microbial communities can be selected to conduct pot experiments and systematically evaluate their promoting effects on the disease resistance, physiological indicators and biomass accumulation of rapeseed under artificial controlled biotic and abiotic stress conditions. Finally, the verified effective strains or synthetic microbial community formulations can be developed into seed coating agents or soil inoculation agents, and their stability and application effects can be further evaluated in field plot experiments. Through the above step-by-step screening and verification process, the ecological findings of this study can be accurately transformed into practical and valuable microbial preparations for rapeseed.

However, this study still has some limitations. Firstly, the inference of microbial composition based on transcriptome data has inherent biases, including the inability to effectively distinguish live bacteria from dead bacteria, as well as underestimation or omission of uncultivable and extremely low-active microorganisms. For instance, based on the analysis of 218 transcriptome samples of rapeseed, this study did not detect the presence of genera such as *Ralstonia*, *Sphingomonas*, and *Brevundimonas*. However, in the network analysis, the co-occurrence network of the abiotic stress group included bacteriophages belonging to the *Tequatrovirus* genus. On one hand, the possible reasons for this were related to the taxonomic annotation method. In this study, Kraken 2.33.0 software and a standard database were used for species classification. This database may not have complete coverage of the genomes of plant endophytes. Compared with the traditional method based on full-length amplification of 16S rRNA gene sequencing, the resolution ability of macro-transcriptome short reads at the genus level was limited, which might cause these reads to be classified into higher taxonomic levels or wrongly assigned to closely related genera. On the other hand, some genera may come from specific rapeseed varieties, geographical regions, or growth conditions, while the 218 public transcriptome samples used in this study may come from different genetic backgrounds or environments. Another possibility was that under certain conditions, some unconventional microorganisms might briefly exist in plant tissues, but their ecological functions still needed to be verified. Secondly, microbial network analysis is based on correlations and lacks in vivo functional validation. This study reveals the positive and negative interactions between microbial genera through co-occurrence network analysis and infers cooperative or competitive relationships based on literature reports. However, correlation is not equivalent to causality or direct biological interactions. The co-occurrence or mutual exclusion between two microbial communities may be driven indirectly by third-party microorganisms, host plant physiological states, or environmental factors, rather than their direct physical or metabolic interactions. Finally, the sample sources and treatment conditions of this study are limited. The observed microbial community composition and network structure may have specific temporal and species-specific characteristics. For example, the genetic background of different rapeseed varieties, the initial load of endophytic bacteria in seeds, and maternal effects, as well as different soil types, climate regions, and other factors may significantly affect the microbial community composition and network structure. Furthermore, some genera enriched on the seed coat surface, such as *Sphingomonas*, may have been effectively removed if the seeds in the original study underwent strict surface disinfection treatment.

These limitations indicated the technical limitations of conducting microbiome research based on public transcriptome data. Therefore, in this study, the microorganisms detected are collectively referred to as the seed-associated microbiome, which includes the epiphytic microorganisms on the seed coat surface, the endophytes inside the seed, and the contaminants introduced during sample processing. In the future, on the one hand, it is necessary to combine 16S/ITS amplicon sequencing and metagenomics methods to verify the same or independent sample sets to distinguish real biological differences from technical deficiencies. It is also necessary to combine macrogenomic sequencing and single-cell sequencing technologies to distinguish live and dead bacteria and conduct a more comprehensive assessment of cryptic microorganisms with low activity but potential functions. On the other hand, isolating and culturing some missing typical endophytes from the original samples and confirming their actual abundance in the samples through qPCR or targeted sequencing will be the key experimental approach to addressing some of the limitations. Additionally, the key bacteria for network prediction that truly occur direct adhesion, signal exchange, metabolite exchange, or antagonism in rapeseed tissues need to be verified in vivo in plants or sterile systems through synthetic microbial communities, co-culture experiments, etc., to lay a theoretical foundation for the final development of precise and efficient rapeseed microbial preparations.

In summary, this study is the first to compare the composition and relative abundance differences of the seed-associated microbiome in rapeseed under development, biotic stress, and abiotic stress. The results in this study should be regarded as an exploratory finding. Future research should combine surface sterilization treatment, negative controls, amplicon sequencing, metagenomics, and cultivation genomics to systematically verify the key conclusions of this study.

## 4. Materials and Methods

### 4.1. Transcriptome Data

The RNA-seq data of 218 published rapeseed seeds were downloaded from the rapeseed multi-omics database (BnIR; http://yanglab.hzau.edu.cn/BnIR; accessed on 25 May 2025). The datasets were in SRA format, comprising 218 files with a total size of approximately 1.39 Tbytes.

### 4.2. Microbial Composition Analysis

Using sratoolkit (v2.9.6), SRA files were converted into Fastq format. The raw sequencing data were then quality-filtered with Fastp (v0.23.4) under default parameters to generate high-quality reads.

Subsequently, SortMeRNA (v4.3.6) was used to separate the transcriptome data into two different files: rDNA sequences and non-rDNA sequences. The obtained rDNA sequence file contained rDNA from both the plant and microorganisms. These rDNA files were processed using Kraken2 [38] to generate a comprehensive species composition. The sequences were compared with the pre-built Kraken2 species composition database using the kraken2 software. The Kraken2 standard database used in this study was constructed on 10 March 2024, and it includes bacteria, archaea, fungi, protozoa, viruses and plasmids. The species classification composition was statistically displayed using the Pavian R package, and the microbial species composition analysis and species abundance information statistical analysis were conducted using the braken software [38], with the classification abundance spectrum refined to the levels of domain, kingdom, phylum, class, order, family, genus, and species. Finally, species-abundance patterns were visualized with stacked bar charts generated using the ggplot2 package (v3.4.0) in R (v4.3.2). To visualize the overlap among different datasets, UpSet plots were generated using the Biozeron Cloud Platform (http://www.cloud.biomicroclass.com; accessed on 31 October 2025).

### 4.3. Diversity and Biomarker Analysis

Alpha diversity metrics, including species richness and Shannon diversity, were calculated using R (v4.3.2). Differences in alpha diversity among the three groups were evaluated by one-way analysis of variance (ANOVA) to test for overall group effects, followed by Tukey’s Honestly Significant Difference (Tukey HSD) test for multiple pairwise comparisons. Statistical analyses were conducted using the alpha_boxplot function implemented in the amplicon R package.

Beta diversity analysis was performed to evaluate the variation in community structure across different ecological systems [39]. To further explore group-specific differences, Partial Least Squares Discriminant Analysis (PLS-DA) was conducted using the mixOmics package. Specifically, supervised PLS-DA was applied at the genus level to model microbial abundance profiles and visualize the dissimilarity between samples, thereby highlighting significant shifts in microbial community composition across the groups. Differential taxa across treatments were identified as potential biomarkers via LEfSe, with an LDA score threshold of 3.5.

### 4.4. Microbial Co-Occurrence Network Construction

Low-abundance endophytic microbial taxa were first filtered out, retaining only those with a relative abundance greater than 0.1% and detected in at least three samples within the abiotic, biotic, and developmental groups. Pairwise Spearman correlation coefficients among the remaining taxa were calculated using the corr.test function in the R package psych. Corresponding *p*-values were adjusted for multiple testing using the Benjamini–Hochberg false discovery rate (FDR) method. Correlation strength was classified according to the absolute value of the correlation coefficient: |r| ≥ 0.7 indicated strong correlation, 0.5 ≤ |r| < 0.7 moderate correlation, 0.3 ≤ |r| < 0.5 weak correlation, and |r| < 0.3 no correlation. Endophytic microbial genera exhibiting significant correlations (|r| > 0.6 and adjusted *p*-value < 0.05) were retained for network construction. Co-occurrence networks for the abiotic, biotic, and developmental groups were constructed and analyzed using Gephi 0.9.2 software. Key topological properties, including degree, weighted average degree, network density, modularity index, average clustering coefficient, and average path length, were calculated to characterize network structure and complexity. These metrics were used to evaluate both within-group and between-group interaction patterns. Network visualization was performed in Gephi 0.9.2. In addition, Cytoscape (version 3.9.1) with the MCODE plugin was employed to identify network modules, enabling the assessment of interaction patterns and differences among endophytic archaea, bacteria, fungi, and viruses across group-specific networks.

## 5. Conclusions

Among the 218 rapeseed samples, the content of fungi and bacteria was the highest, and there were significant differences in the composition and relative abundance of the seed-associated microbiome among different rapeseed samples. The symbiotic networks of the seed-associated microbiome in rapeseed samples for different periods showed obvious morphological differences. Under abiotic stress, a simplified survival mode was manifested. The survival mode under biotic stress and during growth and development stages was more dense and more complex, mainly composed of bacteria–fungal interactions. The upset analysis and alpha diversity showed that the number of unique species and diversity of microorganisms under biotic stress were the highest. Since the seed-associated microbiome offers superior plant compatibility and colonization ability, they can be high-throughput screened for antagonistic or repellent effects against major rapeseed diseases and pests to develop seed coating agents or soil inoculants.

## Data Availability

The original contributions presented in this study are included in the article/Supplementary Material. Further inquiries can be directed to the corresponding authors.

## Supplementary Materials

The following supporting information can be downloaded at: https://www.mdpi.com/article/10.3390/ijms27135801/s1.
